# Synthesis of TiO_2_ nanoparticles and demonstration of their antagonistic properties against selected dental caries promoting bacteria

**DOI:** 10.12669/pjms.39.5.7851

**Published:** 2023

**Authors:** Afsheen Mansoor, Mazhar Mehmood, Muhammad Ishtiaq, Asif Jamal

**Affiliations:** 1 Afsheen Mansoor, Associate Professor, School of Dentistry, Shaheed Zulfiqar Ali Bhutto, Medical University, Islamabad, Pakistan. Quaid-i-Azam University, Islamabad, Pakistan; 2 Mazhar Mehmood Professor, Pakistan Institute of Engineering and Applied, Sciences, Islamabad, Pakistan; 3 Muhammad Ishtiaq Associate Professor, Quaid-i-Azam University, Islamabad, Pakistan; 4 Asif Jamal Associate Professor, Quaid-i-Azam University, Islamabad, Pakistan

**Keywords:** Antimicrobial activity, Bacterial strain, Physicochemical characteristics, Titanium-oxide nanoparticles (TiO_2_Nps), Hydrothermal synthesis

## Abstract

**Objective:**

To evaluate antagonistic role of titanium oxide nanoparticles against selected dental caries promoting bacteria.

**Methods:**

This in vitro-experimental study was conducted at Pakistan Institute of Engineering and Applied Sciences (PIEAS), National Institute of Health (NIH) and School of Dentistry (SOD), Islamabad for the period of one year from February 2022 to January 2023. Modified hydrothermal heating method was used to prepare titanium oxide nanoparticles (TiO_2_Nps). Size, shape, phase, band gap energy, surface and elemental composition of Nps were deciphered by application of various modern techniques including x-ray diffraction spectroscopy (XRD), scanning electron microscopy (SEM), dynamic light scattering (DLS), UV-Vis diffuse reflectance spectroscopy (DRS), atomic force microscopy (AFM), energy dispersive x-ray spectroscopy (EDX). Antimicrobial action of nanoparticles was evaluated against representatives of gram-positive (mono-derm) and Gram negative bacteria (di-derm) responsible for promoting dental caries. The zones of inhibition were calculated by disc diffusion method for each bacterial strain.

**Results:**

Characterization revealed that TiO_2_Nps were having an average size of 54nm, showing anatase-rutile phase having spherical, with very few- irregularly shaped particles. TiO_2_Nps contained only pure titanium and oxygen in the EDX image but organic compounds in FTIR scan. Results of antimicrobial action indicated their potent bactericidal action against *Pseudomonas aeruginosa (20mm), Escherichia coli (19mm) and Lactobacillus acidophilus (19nm)* while comparatively less activity against *Staphylococcus aureus (16mm)*..

**Conclusion:**

TiO_2_Nps fabricated by modified protocol displayed an effective antimicrobial activity and can be used as an alternative to the contemporary chemotherapeutics against selected bacterial pathogens to prevent dental caries.

## INTRODUCTION

The search for novel antimicrobial agents against dental caries promoting pathogens has been a frontier of medical science. Since decades *Streptococcus mutans* have been considered as the initiator of dental caries but many bacteria such as *E. coli, P. aeruginosa* and *S. aureus* are responsible for promoting this oral disease along with other serious infections in humans. Recently, strains of *Lactobacilli* are becoming pathogens particularly in children for causing tooth decay in turn leading to malfunctioning of tooth.^1^ Several investigators posit the presence of different strains of *lactobacilli* in the oral cavity and their association with dental carries.^1^ However, there is quite a limited data regarding antimicrobial action of nanoparticles against different strains of bacteria especially *Lactobacillus*.

Nanotechnology is emerging as one of the most powerful tools for addressing various clinical issues including infectious diseases. The metal oxide nanoparticles have demonstrated excellent antimicrobial potential because of their supramolecular design and ability to interact with biological structures such as cell membrane and associated proteins.^2^ Generally, the nano size and working ability of nanoparticles at atom and molecule level, makes them quite relevant for broader applications in pharmaceutical, health care systems, production of innovative materials, sensor design and in electronics.^3^

In recent years, structural and functional versatility of metal oxide Nps have made them extraordinarily attractive in fields of medicine and dentistry with enhanced antimicrobial activity against variety of bacteria. The TiO_2_Nps are considered as strong antimicrobial agents for bacteria, fungi, and parasites because of their higher biological activity which is associated with generation of increased reactive oxygen species (ROS).^4^ These ROS releases different active radicals and peroxides such as OH^-^, H_2_O_2_ and O^-^ that destroys DNA, protein, cell membrane, cell wall and organelles of pathogenic bacteria subject to their interaction with different biointerfaces.^5^ TiO_2_Nps possess low thermal conduction with exceptional resistance to abrasion, attrition, fatigue, corrosion, tarnish and wear in the oral cavity that makes them suitable for multiple dental applications. Furthermore, these Nps are least toxic and non-allergic in nature displaying acceptable biocompatibility^6^, still they pose threat to human safety^7^, by reducing their antimicrobial sensitivity against certain strong bacterial strains. Currently, these bacteria have developed innovative resistance mechanisms against the prevailing antimicrobials, thus imposing greater threat for public health.^5^,^8^

The biological and physicochemical properties of Nps responsible for antimicrobial activities may include their size, shape, phase, surface roughness, band gap energy, morphology, elemental composition and functional compounds.^9^ Different types of fabrication protocols for Nps influence their physicochemical characteristics,^10^ that might have an adverse effect on their antimicrobial activity against bacteria. Nps are fabricated by incorporating biogenic protocols (bacteria, algae, fungi, parasite, plants) and conventional protocols (chemical & physical).^10^ Although, literature reported the stable and sustainable behavior of TiO_2_Nps generated by biological route,^11^ but quick production of these Nps with uniformity and purity was observed after following the conventional route.^12^ The high level of pressure, temperature, chemical accelerators and energy consumption in the conventionally fabricated Nps might have a negative impact on both the humans and environment. There is a variety of conventional methods used for the synthesis of TiO_2_Nps such as photochemical processes, laser ablation, solvo-thermal, radiation, physical, chemical, pyrolysis, radiation, and electrochemical processes.^11^ However, hydrothermal processes for TiO_2_Nps synthesis are considered more appropriate because of certain added advantages. One of the limitations of this process is use of high pressure and temperature for making NPs that could have adversely affected the bactericidal activities.

Moreover, high temperature is linked with Nps size thus, influencing its bactericidal properties.^13^ There is a dire need to achieve controlled Nps with desirable size that could be attained by reduction in the process temperature. This modification in synthesis protocol could effectively enhance the bactericidal activities by controlling NPs size. Therefore, the current study aimed to produce novel TiO_2_Nps with help of modification in conventional hydrothermal heating process at comparatively low temperature in order to address NPs size related problems. The prepared TiO_2_Nps were characterized by state of art analytical techniques such as XRD, DRS, AFM, SEM, EDX, FTIR and DLS. Further, TiO_2_Nps were evaluated for their antimicrobial activity against both Gram-negative and Gram-positive bacterial strains to demonstrate their possible role as alternative antibacterial agents to prevent dental caries in future.

## METHODS

This in vitro-experimental study was conducted at Pakistan Institute of Engineering and Applied Sciences (PIEAS), National Institute of Health (NIH) and School of Dentistry (SOD), Islamabad for the period of one year from February, 2022 to January 2023, after the ethical approval (Approval Letter # SOD/ERB/2023/22) from School of Dentistry, Shaheed Zulfiqar Ali Bhutto Medical University, Islamabad, Pakistan.

### Inclusion criteria:

Standard bacteria strains with ATCC number were used in the study which included:

- *S. aureus* (ATCC®25923™),
- *E. coli* (ATCC®35218™),
- *L. acidophilus* (ATCC®314™),
- *P. aeruginosa* (ATCC®27853™).

### Exclusion criteria:

- Bacterial strains without having ATCC number were excluded from this study.

Synthesis of NPs was carried out by modification in existing standard protocol mentioned in literature.1 mL TiCl_4_ salt (Sigma-Aldrich, Merck KGaA, DS, Germany) was dissolved in 100ml of de-ionized water to make 1 molar salt solution of TiCl_4_. It was placed on hot plate (WITEG. Model: MSH-20A. P/N: DH.WMH03120, MA, Burma), and heated at 100^o^C until color of solution changed from black to white that confirmed presence of TiO_2_Nps which were centrifuged till white jelly like cake was formed. It was then subjected for drying in an autoclave at 100^o^C. Finally, this dried cake was calcinated at 500 C^o^ to get fine-powdered form.^14^ The characterization of these Nps was carried out by XRD (DPP-MAXZ ,24000-Diffractometer, Rigakuu Corporation’s, Akishimia, Tokyo; Japan), DLS (Zeta-sizer Nano Z/SS Apparatus-; ZENN’s 36000, Malvernnpanalytical’s, Malvernn; UK), SEM & EDX (Nova nanosemnn 430, FEIII company 40222-261, 49391 SS column F/G’s strons prep-p, Hillsboro, OR; USA), AFM (Quesants Universal SSPMs, Ambioos Technology, Santaa Cruz-z, CA; USA) and FTIR (JAASCO-1 FTIRr-6600s, Ultrech’s Amsterdam, AMSS; Netherlands).^10^

The prepared TiO_2_Nps were tested against selected bacterial pathogens for the investigation of antimicrobial sensitivity using disc diffusion method. The 3.8 Grams of agar powder (Muller Hinton), was added to 100 Milliliters distilled water which was kept in autoclave at 15.0lbs for 15min at 121^o^C and was poured into Petri plates. Bacterial isolates were kept in peptone-broth for 3-4 hours after incubating them at 37^o^C to get bacterial suspensions. After dipping sterile cotton swab in bacterial culture suspension, it was streaked three times on same sterile Petri plates specific for same bacterial strain. The 250µg/L TiO_2_Nps suspension was prepared by dissolving them at a ratio of 1mg/mL in distilled water. Then, diameter of 3mm Whatmann paper was dipped into Nps suspension and placed on bacterial culture streaked on each petridish. The plates were then incubated for twenty-four hour at 37^o^C in order to observe inhibitory zones.^15^

## RESULTS

**Fig.1:**
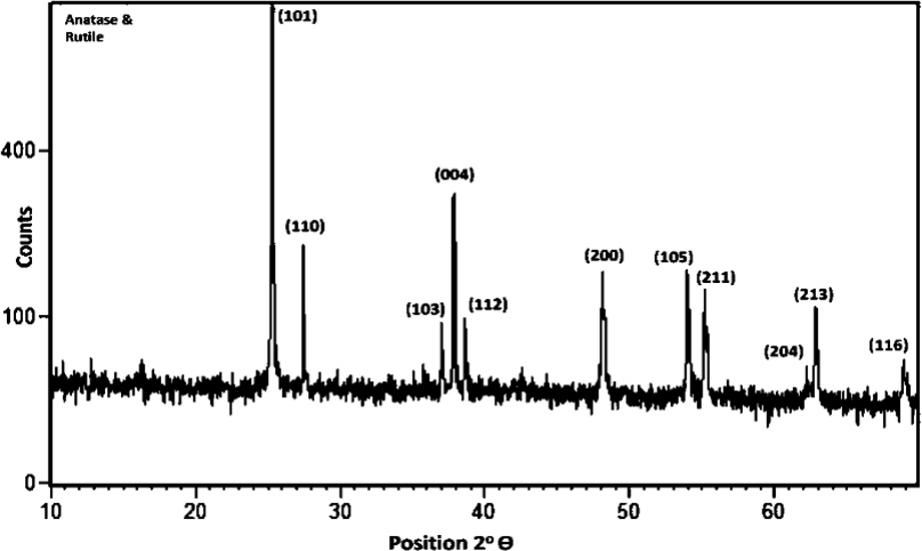
XRD pattern of TiO_2_Nps depicting various peaks referred to anatase-rutile phases.

**Fig.2:**
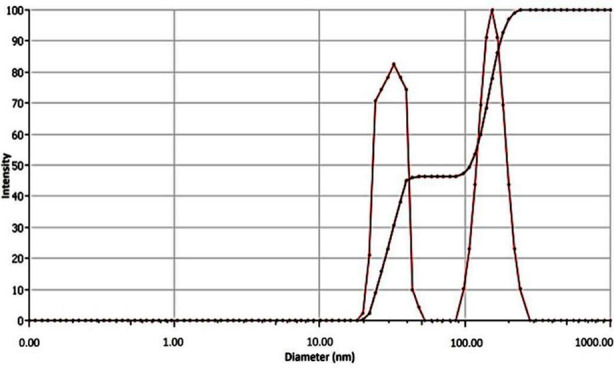
DLS spectrum, of TiO_2_Nps demonstrating average particle size.

TiCl_4_ substrate solution was black in color before experiment and changed to white color after experiment confirming presence of TiO_2_Nps. XRD profile of these Nps is presented in Fig.1 and peaks were compared with JCPDS card no 01-071-1166. Results indicated that main peak at 101 represented anatase phase of NPs at 2θ = 25.32°. Other peaks were observed at (103) 37.00°, (004) 37.84°, (112) 38.61°, (200) 48.12°, (105) 53.98°, (211) 55.16°, (213) 62.30°, (204) 62.82° and (116) 68.92°. The only rutile peak observed at (110) 2θ = 27.45◦ was in collaboration with JCPDS card no 01-077-0440. These TiO_2_Nps were found to be in 82% pure anatase phase and 18% rutile phase (Fig.1) having crystalline size of 52.28nm, determined by Debye - Scherrer’s formula. DLS scan showed 58nm particle size of TiO_2_ Nps which was slightly greater than XRD and SEM (Fig.2).

**Fig.3:**
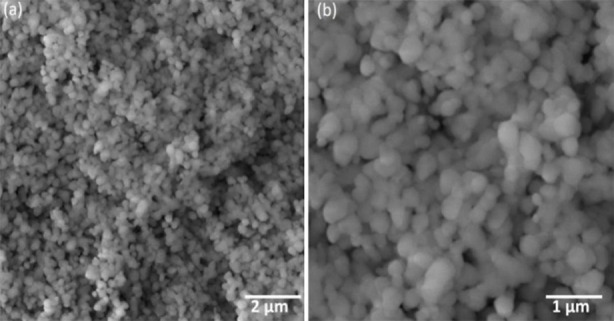
Scanning electron microscopic image of TiO_2_Nps at (a) 20 kx (b) 50 kx showing size and shape.

**Fig.4:**
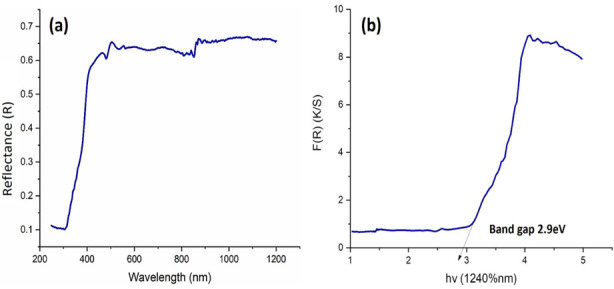
DRS pattern scan of TiO_2_Nps showing: (a) formation, (b) crystalline size.

**Fig.5:**
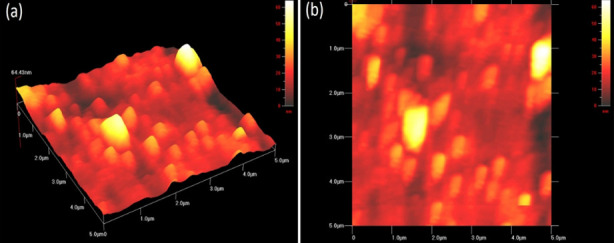
Image of TiO2Nps showing size and spherical shape with few irregularities in: (a)3D horizontal plane (b)2D vertical plane.

**Fig.6:**
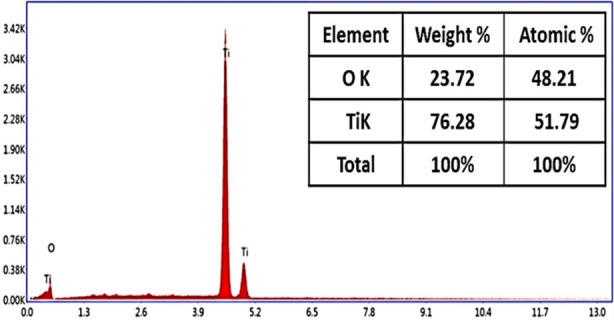
EDX analysis of TiO_2_ Nps displaying presence of titanium and oxygen.

Scanning electron microscopic analysis of TiO_2_Nps revealed their large size of about = 54nm having mixture of spherical and slight (few) irregularly shaped particles (Fig.3). DRS spectrum confirmed formation of TiO_2_ Nps at 337nm wavelength having calculated band energy of 2.9 eV which confirmed their slightly big crystalline size (Fig.4). The mixture of dominantly spherical with slight irregularly shaped particles was observed in the AFM scan of these TiO_2_Nps (Fig.5). Main peaks of tiatanium (Ti) and oxygen (O) were ascertained in EDX spectrum. The weight % and atomic % of Titanium was 76.28% and 51.79% whereas oxygen was 23.72% and 48.21% (Fig.6).

**Fig.7:**
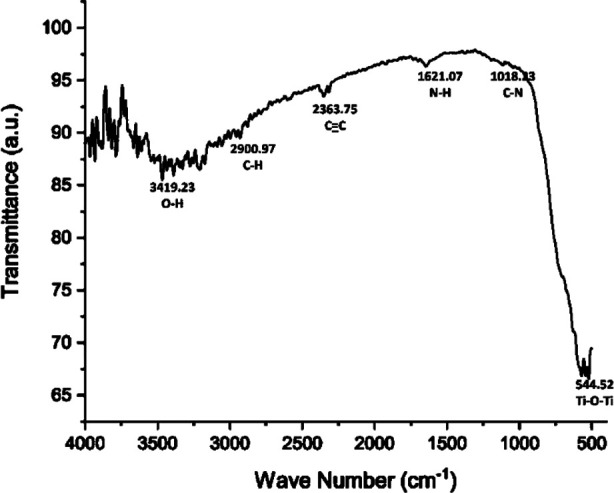
FTIR spectrum, of TiO_2_Nps demonstrating peaks of various functional compounds.

Peaks at 3491.23 cm-^1^, 3277.19 cm-^1^, 3113.41 cm-^1^ and 1679.13 cm-^1^ occurred because of O-H stretching vibrations of alcohol groups, peaks at 2900.97 cm-^1^ revealed C-H stretching of organic compound while peaks at 2363.75 cm-^1^ and 675.11 cm-^1^ attributed to C-C stretching vibrations of alkyne groups. The C-N stretching vibrations of amines were recorded at 1453.71 cm-^1^ and 1018.23 cm-^1^. Other peaks such as 1937.49 revealed C-H bending of aromatic compound, 1679.13 cm-^1^ peak showed O-H bending of hydroxyl group, 1621.07 cm-^1^ peak revealed N-H bending of amine group, 1557.91 cm-^1^ peak observed N-O stretching of nitro-compound. The last peak was evident at 540.52 cm-^1^ ensuring Ti-O-Ti bending resulting inTiO_2_Npsformation (Fig.7). TiO_2_Nps revealed maximum antimicrobial activity through zone of inhibition against *E.c oli* and *P. aeruginosa*, while comparatively less against *S. aureus* and *L. acidophilus* (Table-I).

**Table-I:** Zone of Inhibition produced by TiO_2_Nps against gram negative and gram-positive bacterial strains.

Sr.	Bacterial Strain	Zone of Inhibition (mm) produced by TiO_2_Nps synthesized by modified Conventional hydrothermal heating
1.	*Pseudomonas aeruginosa*	20 ± 1.5
2.	*Lactobacillus acidophilus*	19 ± 1.5
3.	*Staphylococcus aureus*	16 ± 0.7
4.	*Escherichia coli*	19 ± 1.5

## DISCUSSION

The modification in conventional hydrothermal heating synthesized novel TiO_2_Nps revealed presence of mixed anatase-rutile phase with 52.28nm size (XRD), hydrodynamic size of 58 nm (DLS), mixture of dominantly spherical with slightly irregular shaped particles having size of about 54 nm (SEM and AFM), band gap energy of 2.9 ev (DRS), pure titanium and oxygen peaks (EDS) with several functional groups (FTIR). These TiO2 Nps displayed an acceptable antimicrobial activity, (CLSI recommendations), against different dental caries promoting bacterial strains such as *P. aeruginosa, E. coli, L. acidophilus* and *S. aureus*. First, change of titanium tetrachloride solution’s color from black to white confirmed formation of TiO_2_Nps.

This observation could be linked to oxidation reduction process resulted in change of titanium tetra chloride solution color under influence of processing temperature and pressure.^16^ Previously, M. Challapa et. Al. reported that size, shape, phase form, surface roughness, elemental composition and functional compounds regarding Nps are key indices that confirm their medicinal and therapeutic effectiveness.^17^ The TiO_2_ Nps in this study confirmed presence of 82% anatase phase and 18% rutile phase with 52.28nm size in XRD spectroscopy (Fig.1), hydrodynamic size of 58 nm in DLS (Fig.2) and mixture of spherical and irregular shaped particles having size of about 54 nm in SEM (Fig.3). The DRS spectroscopy confirmed band gap energy whose standard value of 3.2ev is also related to the size. The band gap energy value in this study is less than standard value which is an indicator of this particular particle size of Nps obtained (Fig.4).^18^

The formation of TiO_2_Nps at the wave length of 337nm matched the literature where these Nps were formed at wave length of 337 nm.^19^ The AFM image confirmed presence of nanosized particles having mixture of predominantly spherical with slightly irregular shape (Fig.5). Contrary to previous reports, particularly with reference to size of NPs, the size of TiO_2_Nps in our study was comparatively smaller. In a study, size of TiO_2_Nps was found to be 72.55nm while using high temperature for their synthesis.^14^ Therefore, low temperature strategy employed in our study was proved effective and supported the hypothesis of using low temperature for reducing size and biological performance of NPs.

The EDX spectroscopy displayed presence of titanium and oxygen in its composition without involvement of any other element, which might have enhanced their efficacy (Fig.6). On the other hand, FTIR spectroscopy confirmed presence of large amount of functional compounds in spectrum of these newly formed TiO_2_Nps (Fig.7).^20^ The C-H stretching peak at 2900.97cm^-1^ observed in this study is marker of presence of some kind of additional compounds as reported by Augugliaro V et al.^21^ The TiO_2_Nps produced by modified Conventional hydrothermal heating have employed chemical activator at lower temperature and pressure in this study that might have resulted in production of large amounts of different functional group chains. The presence of these additional compounds could challenge the biocompatibility of these Nps in future and further studies are required to address this.

The current study displayed an effective inhibitory activity of TiO_2_Nps against *P. aeruginosa E. coli, L. acidophilus* and *S. aureus* (Table-I). The antimicrobial activity of TiO_2_Nps against gram negative bacteria was more as compared to Gram-positive bacteria due two possible reasons: (a) cell wall of gram negative bacteria is composed of thin peptidoglycan layer which might be easily dissolved by these Nps,^22^ (b) difference of charge between these Nps (+ve) and bacterial strains (-ve), might have enhanced force of attraction between them resulting in their oxidation and death.^23^ According to standard protocol, antimicrobial activity is counted as: sensitive (at or > 19mm), intermediate (15-18mm) and resistant (at or < 14mm) which depends on presence of the inhibitory zones.^24^ A study performed by Yadav K et. al confirmed that certain bacteria (*P. aeruginosa, E. coli, L. acidophilus* and *S. aureus)* are considered responsible for various medical and dental diseases in human beings.^1^

The antimicrobial activity of Nps is greatly attributed to their physicochemical properties such as shape, size, surface, synthesis, composition and functional compounds as reported by Pradhan et al.^25^ The findings in the current study matched the work done previously by Priyanka KP et al that prepared TiO_2_Nps through sol gel method.^24^ A study demonstrated the physical and biological properties of FeO nanoparticles, which reported that size of about 52.7 nm nanoparticles displayed excellent antibacterial activity against *E. coli, K. pneumoniae and S. aureus*.^26^ *In another work, bactericidal potential of iron oxide nanoparticles against* S. dysentery, E. coli and S. aureus was *evaluated.^27^*

The titanium and zinc oxide based NPs showed significant activity when tested on *S. aureus, P. fluorescens, L. monocytogenes* and E. coli.^28^ Few other studies have also concluded significant antimicrobial activity of natural ingredients and metal salts against resistant bacterial strains.^29^-^31^ These studies provide compelling evidence regarding metal oxide nanoparticles and their suitability for addressing the challenge of bacterial infections.^32^ *These* TiO_2_Nps depicted significant antimicrobial activity against various bacteria because of modification in temperature employed for their synthesis because ‘synthesis protocols’ are considered responsible for biological properties such as biocompatibility and antimicrobial activity which are considered as the sole requirement for their usage in clinical dentistry.

### Limitations

Further modifications in synthesis protocols of nanoparticles is recommended where eco-friendly materials can be employed for their preparation. Future studies are required to investigate the in-vivo response of TiO_2_ Nps against caries promoting bacteria in the oral cavity.

## CONCLUSION

Modifications in nanoparticles synthesis protocol are quite helpful in attaining the desired size of TiO_2_Nps that could be helpful in enhancing their antimicrobial activity against the caries promoting bacterial strains of *P. aeruginosa, E. coli, L. acidophilus* and *S. aureus*. Based on our results, it could be concluded that Titanium based Nps can be used as a possible alternative to conventional antimicrobial agents in order to prevent dental caries in the oral cavity.

### Author Contributions:

**AM:** Concept, study design, data collection, analysis, interpretation, writing first draft.

**MM:** Data Analysis, interpretation, drafting, critical review of manuscript.

**MI:** Data analysis, evaluation, interpretation, revision of manuscript.

**AJ:** Concept, study design, supervision, Methodology of research project, IRB and final version approval, responsible for integrity of study.
